# Stroma-normalised vessel density predicts benefit from adjuvant fluorouracil-based chemotherapy in patients with stage II/III colon cancer

**DOI:** 10.1038/s41416-019-0519-1

**Published:** 2019-07-10

**Authors:** Artur Mezheyeuski, Ina Hrynchyk, Mercedes Herrera, Mia Karlberg, Eric Osterman, Peter Ragnhammar, David Edler, Anna Portyanko, Fredrik Ponten, Tobias Sjöblom, Bengt Glimelius, Arne Östman

**Affiliations:** 1 Department of Oncology-Pathology, Karolinska Institutet, Stockholm, Sweden; 2 Department of Immunology, Genetics and Pathology, Uppsala University, Uppsala, Sweden; 3 City Clinical Pathologoanatomic Bureau, Minsk, Belarus; 4 Department of Molecular Medicine and Surgery, Karolinska University Hospital Solna, Stockholm, Sweden; 5 N.N. Alexandrov National Cancer Centre of Belarus, Minsk, Belarus

**Keywords:** Colon cancer, Predictive markers

## Abstract

**Background:**

Identification of biomarkers associated with benefit of adjuvant chemotherapy in stage II/III colon cancer is an important task.

**Methods:**

Vessel density (VD) and tumour stroma were analysed in a randomised-trial-derived discovery cohort (*n* = 312) and in a stage II/III group of a population-based validation cohort (*n* = 85). VD was scored separately in the tumour centre, invasive margin and peritumoral stroma compartments and quantitated as VD/total analysed tissue area or VD/stroma area.

**Results:**

High stroma-normalised VD in the invasive margin was associated with significantly longer time to recurrence and overall survival (OS) (*p* = 0.002 and *p* = 0.006, respectively) in adjuvant-treated patients of the discovery cohort, but not in surgery-only patients. Stroma-normalised VD in the invasive margin and treatment effect were significantly associated according to a formal interaction test (*p* = 0.009). Similarly, in the validation cohort, high stroma-normalised VD was associated with OS in adjuvant-treated patients, although statistical significance was not reached (*p* = 0.051).

**Conclusion:**

Through the use of novel digitally scored vessel-density-related metrics, this exploratory study identifies stroma-normalised VD in the invasive margin as a candidate marker for benefit of adjuvant 5-FU-based chemotherapy in stage II/III colon cancer. The findings, indicating particular importance of vessels in the invasive margin, also suggest biological mechanisms for further exploration.

## Background

Improved imaging and surgical procedures, including total mesorectal excision in rectal cancer and mesocolic excision in colon cancer, have dramatically improved outcomes in colorectal cancer (CRC) patients.^1–3^ Despite these successes, recurrences in stage II colon cancer are still seen in up to one-quarter of the patients^3–5^ and in stage III in up to every other patient. Adjuvant therapy is therefore recommended, reducing the risks by about one-third.^6,7^

Patients with resected tumours with metastatic growth in regional lymph nodes (stage III) generally receive treatment after surgical removal.^7^ The benefit of adjuvant chemotherapy in patients with lymph-node-negative disease remains a subject of discussion,^6,8^ and adjuvant chemotherapy is not recommended for most patients with stage II colon cancer.^7,9^ It is generally believed, however that a fraction of stage II group benefits from the adjuvant treatment, and most guidelines recommend therapy for high-risk groups. Criteria for high risk of recurrence include a low number of sampled lymph nodes (<12), low tumour differentiation and T4 stage according to US criteria (https://www.nccn.org/professionals/physician_gls/default.aspx#colon) and vascular invasion, lymphatic or perineural invasion and obstruction (ESMO criteria).^7^ For stage III, a combination of oxaliplatin with a fluoropyrimidine is routinely recommended. However, the generalisation of the addition of oxaliplatin has been questioned in several studies, particularly in patients above 70 years.^10^

Taken together, this situation identifies a need for identification of better criteria for stratification of patients to groups who benefit sufficiently from adjuvant treatment in both stage II and stage III colon cancer,^11,12^ predicting the need for adjuvant treatment, i.e. the presence of subclinical deposits, and the benefit of such chemotherapy, i.e. reducing the number of deposits, leading to a recurrence and ultimate death, is a highly active research area.^13^ Candidate markers subject to ongoing validation include CD133 and MMR status.^14–16^

A potential impact of tumour vessel characteristics on drug delivery and sensitivity to chemotherapy is suggested by experimental studies.^17–20^ Recent studies also imply tumour vasculature as an important component of cellular niches harbouring chemo-resistant cancer stem cells.^21–24^ Properties of the vasculature also affect cancer cell intravasation and establishment of distant metastases.^25–30^ Notably, while earlier studies on prognostic or response-predictive significance of vessel density (VD) have mostly used semi-quantitative visual scoring procedures, recent studies have taken the advantage of automated digital-image analyses for more observer-independent determinations of vascular features.^20,31–33^

In this study, we use CD34 as a vessel marker and PDGFR-β as a marker for tumour stroma. This study has explored the potential predictive significance of VD in two well-annotated clinical cohorts. The study thereby goes beyond earlier studies firstly, through separate analyses of different anatomical regions, and secondly, by distinct analyses of total tumour VD and stroma-normalised VD.

## Methods

### Clinical data and study cohorts

Two independent collections of a surgically resected material of colon cancers were used. For the *Discovery cohort*, we used tissue material derived from 312 patients from a Nordic randomised clinical trial performed to evaluate the efficacy of 5-FU (5-fluorouracil)-based adjuvant chemotherapy.^34^ The study included 2224 patients younger than 76 years with radically resected stage II–III colorectal cancer operated during the time period 1991–1997. These patients were randomised to either surgery alone or surgery followed by adjuvant chemotherapy. The chemotherapy regimens included 5-FU/leucovorin for 4–5 months, according to either a modified Mayo Clinic or the Nordic schedule or 5-FU/levamisole during 12 months. No patient received radiotherapy or chemotherapy prior to the surgery. Tissue from surgical resection was used. Fresh sections were cut for the study.

The ethical committee of the Karolinska Institutet, Stockholm, Sweden, approved the analysis (Dnr 00-260, 2014/664-32).

As a *Validation cohort*, we used colon cancer tissue microarray (TMA) derived from a population-based CRC collection obtained in the context of U-CAN, Sweden (http://www.u-can.uu.se/about-u-can/).^35^ U-CAN patients from the county of Uppsala (Sweden) diagnosed with stage II/III colon cancer between 2010 and 2014 who had radical surgery and survived at least 6 weeks after surgery were included in the validation cohort. Patients were treated within routine care with adjuvant therapy, according to ESMO guidelines. TMAs were made from formalin-fixed paraffin-embedded tissue blocks of primary tumour. Each case is represented on the TMA with two cores derived from the central part of the tumour and two cores from the invasive margin. No rectal cancer cases were included in the study.

The regional ethical committees in Uppsala, Sweden approved the analysis (Dnr 2010/198 and Dnr 2015/419).

### IHC procedures

Four-micrometer-thick sections were de-paraffinised, rehydrated and rinsed in distilled H_2_O. The antigen retrieval with boiling in pH 10.0 retrieval buffer was performed in decloaking chamber (Biocare Medical) at 110 °C for 5 min. Sections were then incubated with blocking solution for 30 min and with PDGFR-β rabbit monoclonal antibody (#3169, Cell Signaling Technology, Danvers, MA), 2 µg/ml at dilution 1:100 overnight. The sections were incubated with the polymer system (ImmPRESS™-AP Polymer Anti-Rabbit IgG MP-5401) for 1 h at room temperature and developed with Vector^®^ Blue AP Substrate Kit (SK-5300, Vector Laboratories, Burlingame, CA). To inactivate alkaline phosphatase reagents, the sections were heated in decloaking chamber at 95 °C for 5 min, in pH 9.0 solution. This was followed by an incubation with blocking solution for 30 min and with anti-CD34 (Clone JC70A; Dako, Inc., Denmark) at dilution 1:100 overnight. Sections were then incubated with polymer system (ImmPRESS™-AP Polymer Anti-Mouse IgG, MP-5402, Vector Laboratories, Burlingame, CA) for 1 h at room temperature, and developed with Vector^®^ Red AP Substrate Kit (SK-5100, Vector Laboratories, Burlingame, CA).

### Quantitative analysis of immunohistochemical staining

The double-stained slides of the discovery cohort (regular sections) were scanned by a V-slide-scanning microscope (Metasystems, Alltlussheim, Germany), with × 10 objective and RGB-led illumination for colour deconvolution. The Metaviewer (Metasystems, Alltlussheim, Germany) was used to view the scanned digital slides. Each tumour sample was reviewed by the same pathologist (IH) and three regions/compartments were selected: tumour centre, invasive margin and peritumoral stroma.

The region selection was morphology-based and was made with the intention to capture as big area as possible. For the invasive margin, the tumour region facing the adjacent non-malignant tissue was selected. The rest of the tumour mass was considered as tumour centre. Peritumoral non-malignant tissue was only selected if characterised by fibrosis. Non-malignant fat tissue or muscle tissue was not included into selection. In all three compartments, the regions without necrosis and artefacts was selected. Small artefacts were removed manually.

All cases were reviewed by the second pathologist (AM). A joint decision was made in conflicting cases. The region selection is schematically illustrated in Supp Fig. 1a. These compartments were annotated and saved in .tif format as individual images.

**Fig. 1:**
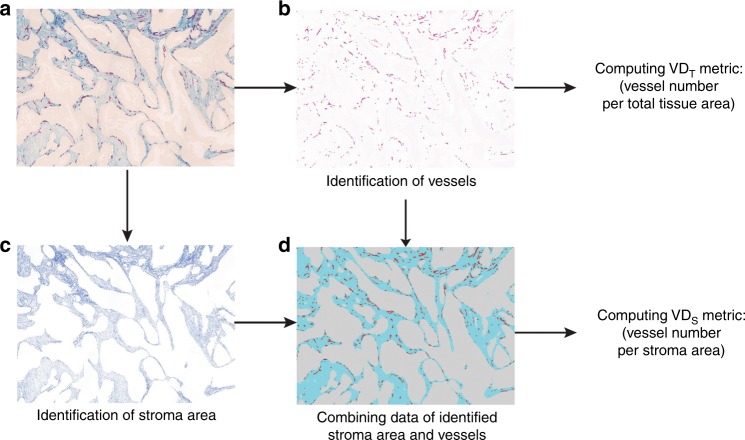
Determination of the ‘VD_T_’ and ‘VD_s_’ metrics. The original image was double stained for vessels with an antibody against CD34 (red) and for stroma with a PDGFR-β antibody (blue) (**a**) and was used to quantify vessels (**b**). The PDGFR-β-positive sub-regions, defining the stromal area, are illustrated in (**c**). To obtain ‘VD_T_’ values, the number of vessels per total tissue area was determined (**b**). To obtain ‘VD_S_’ values, the number of vessels per stroma area was determined (**d**)

The double-stained TMA slides of the validation cohort were scanned by Aperio Scanscope AT with × 20 objective. Pictures corresponding to the individual TMA cores were extracted and saved in .tif format as individual images. Most of the cases in this cohort were represented by two cores from tumour centre and two cores from the invasive margin. The cores of the same origin were treated for image analysis as one entire tissue sample.

The images were then used for automated image analyses to define the total tissue area, stromal area and vessel quantity (for details of this methodology see refs. ^33,36^). Vessels were identified by CD34 staining and quantified by an in-house developed image analysis algorithm (ImageJ software). The total tissue area was identified on the pre-selected images, representing the three tumour compartments described above. Blank areas present on the selected regions were excluded from the total tissue area quantification. PDGFR-β-positive regions were considered as stromal (for details see Materials and Methods in ref. ^36^). Vessel quantity and total tissue area were used to compute the ‘**VD**_**T**_’ metric (number of vessels per total analysed tissue area). Vessel quantity and PDGFR-β-positive stroma regions were used to compute the ‘**VD**_**S**_’ metric (number of vessels per stroma area).

For each of these three compartments, two values were calculated: number of vessels per analysed total tissue area (VD_T_) and number of vessels per tumour stroma area (VD_S_), making together six VD metrics per case: **VD**_**T**_^**CT**^**, VD**_**T**_^**IM**^, **VD**_**T**_^**Peri**^**, VD**_**S**_^**CT**^**, VD**_**S**_^**IM**^ and **VD**_**S**_^**Peri**^. Because in the validation cohort only tissue material from tumour centre and invasive margin was available (but not for peritumoral tissue), four metrics from two compartments were produced in the validation cohort: **VD**_**T**_^**CT**^**, VD**_**T**_^**IM**^**, VD**_**S**_^**CT**^ and **VD**_**S**_^**IM**^. The analytical pipeline is schematically illustrated in Fig. 1.

### Tumour budding and configuration of the invasive border

Assessment of the configuration of the tumour border was done according to recommendations of Morikawa et al.^37^ The growth pattern was categorised into pushing (expansile), intermediate or infiltrative. A pushing growth pattern was considered a circumscribed tumour border. An infiltrative pattern was characterised by the presence of irregular clusters, small islands of cancer cells or small glands without a distinct border in the area of the invasive front. Appearance of the large and medium-sized glands at the invasive border was linked to the intermediate growth pattern. The assessment was performed independently by a pathologist (AM) and a clinical researcher (MK) after extensive training. In all cases of discrepancy in the scoring between the two observers, a collegial decision was made.

Tumour budding evaluation was performed, using as a model the guidelines of Karamitopolou et al.^38^ The trained pathologist (AM) identified ten fields of view on the digitalised slides, along the area of maximal invasion of the tumour. The area of the fields corresponded to the high-power field area of the microscopes used for the diagnostic routines. The number of tumour buds was counted by two independent observers (AM and MK) in each area and the average value was calculated. It was then used to dichotomise cases into high-budding (having ≥ 10 buds) and low-budding (<10 buds) groups. All cases with inter-observer difference in the final budding score were reviewed and a collegial decision was made.

### Statistical analyses

Statistical analyses were carried out using SPSS V20 (SPSS Inc., Chicago, IL) and R software, version 3.3.3 (R Core Team (2017)). R: a language and environment for statistical computing. R Foundation for Statistical Computing, Vienna, Austria. URL (https://www.R-project.org/), and integrated development environment RStudio, version 1.0.143 (RStudio Team (2015). RStudio: Integrated Development for R. RStudio, Inc., Boston, MA, URL http://www.rstudio.com/) with the following packages: gdata, ggplot, corrplot, PerformanceAnalytics, scales, rms, survival, made4, reshape, plyr and maxstat.

Time to recurrence (TTR) was computed as the time from surgery to the first documented disease progression, including local recurrence or distant metastases or death due to colon cancer, whichever occurred first.^39^ Overall survival (OS) was the time from surgery to death due to any reason. To estimate relative hazards in both univariate and multivariable models, a Cox proportional hazards model was used. For the analyses of associations between VD and clinical characteristics, chi-square test was used.

All statistical tests were two-sided and *P*-values < 0.05 were considered statistically significant. The correction for the multiple testing was applied in the exploratory part of the study, using the ‘BH' (aka ‘fdr') method of Benjamini, Hochberg and Yekutieli, and reported as ‘q-values’.

## Results

### Quantitative characterisation of six vessel-density-related features in a randomised-trial-derived colon cancer collection

The colon cancers from the Nordic adjuvant randomised clinical trial,^34^ investigating benefits of 5-FU-based chemotherapy in stage II/III CRC, have been used in previous studies for the identification of prognostic and predictive markers.

In this study, this collection was used as a discovery cohort to explore potential relationships between VD-related features and benefit of chemotherapy. For each of these compartments, two values were calculated: number of vessels per analysed total tissue area, including both tumour and stroma compartments (VD_T_) and number of vessels per tumour stroma area only, referred further in the text as ‘stroma-normalised VD’ or abbreviated as ‘**VD**_**S**_’. By this approach, we generated together at maximum six VD metrics per case (see Methods and Fig. 1).

Successful staining was obtained on 312 of originally 514 tumours. The Digital image analyses-derived data were collected as follows: 282 cases from the tumour centre, 285 cases from the invasive margin and 176 cases from the peritumoral stroma (see Supplementary Fig. 1B). The high rate of the case loss is explained by tissue damage and detachment during the antigen retrieval procedure. The number of the cases with analysed peritumoral stroma was also limited by the presence of fibroblastic tissue available for analysis (see Methods for more detail). To control potential selection bias, all three subpopulations were compared with regard to clinico-pathological characteristics, with no statistically significant difference observed (Supplementary Table 1), and treatment subgroups were compared in each of three subpopulations (Supplementary Table 2). In addition, we performed a comparative analysis of the survival of three subpopulations, sub-divided by treatment. No statistically significant differences were found in TTR or OS between the surgery-alone group and the group having surgery followed by adjuvant chemotherapy (Supplementary Fig. 2). These results are similar to the overall results in the original study population, revealing a statistically non-significant absolute gain of 8% in OS in colon cancer stage III only.^34^

Initial analyses demonstrated significant differences between the three tumour compartments, regarding both **VD**_**T**_ and **VD**_**S**_ (Supplementary Fig. 3). VD_T_ was the highest in peritumoral stroma regions, whereas **VD**_**S**_ was higher in the tumour regions than in the surrounding peritumoral stroma. Additional analyses demonstrated moderate within-case pairwise associations of **VD**_**T**_ and **VD**_**S**_, indicating the possibility that these metrics could capture different aspects of vessel biology (Supplementary Fig. 4). Interestingly, higher **VD**_**S**_ in tumour centre and in the invasive margin was inversely associated with adverse factors, such as infiltrative configuration of the invasive border and high-budding score (Table 1).

**Table 1:** Associations between VD in three tumour compartments and clinico-pathological characteristics in the discovery cohort

	Tumour centre			Invasive margin			Peritumoral stroma		
	VD_S_^CT^ number (percent)			VD_S_^IM^ number (percent)			VD_S_^Peri^ number (percent)		
Characteristic	Low	High	*p*	Low	High	*p*	Low	High	*p*
Age (years)									
<66	66 (23.4)	56 (19.9)	0.279	68 (23.9)	62 (21.8)	0.476	41 (23.3)	42 (23.9)	1.000
≥66	75 (26.6)	85 (30.1)		74 (26.0)	81 (28.4)		47 (26.7)	46 (26.1)	
Sex									
Male	69 (24.4)	78 (27.7)	0.340	69 (24.2)	74 (26.0)	0.636	44 (25.0)	43 (24.4)	1.000
Female	72 (25.5)	63 (22.3)		73 (25.6)	69 (24.2)		44 (25.0)	45 (25.6)	
Tumour site									
Proximal^a^	74 (26.2)	75 (26.6)	1.000	80 (28.1)	83 (29.1)	0.811	47 (26.7)	50 (28.4)	0.762
Distal	67 (23.8)	66 (23.4)		62 (21.8)	60 (21.1)		41 (23.3)	38 (21.6)	
Mismatch-repair status									
MMR proficient	108 (41.1)	118 (44.9)	0.216	110 (41.4)	106 (39.8)	0.638	62 (38.3)	65 (10.5)	0.850
MMR deficient	22 (8.4)	15 (5.7)		23 (8.6)	27 (10.2)		18 (11.1)	17 (10.5)	
TS expression									
High	103 (36.5)	110 (39.0)	0.406	106 (37.2)	111 (38.9)	0.581	70 (39.8)	67 (38.1)	0.717
Low	38 (13.5)	31 (11.0)		36 (12.6)	32 (11.2)		18 (10.2)	21 (11.9)	
Stage									
II	57 (20.2)	60 (21.3)	0.809	51 (17.9)	66 (23.2)	0.092	32 (18.2)	41 (23.3)	0.221
III	84 (29.8)	81 (28.7)		91 (31.9)	77 (27.0)		56 (31.8)	47 (26.7)	
Adjuvant chemotherapy									
Yes	71 (25.2)	72 (25.5)	1.000	77 (27.0)	73 (25.6)	0.636	47 (26.7)	44 (25.0)	0.763
No	70 (24.8)	69 (24.5)		65 (22.8)	70 (24.6)		41 (23.3)	44 (25.0)	
Invasive border configuration									
Pushing	32 (12.2)	64 (25.2)	<0.001	30 (11.7)	69 (26.8)	<0.001	29 (18.1)	43 (26.9)	0.098
Intermediate	29 (11.4)	29 (11.4)		24 (9.3)	34 (13.2)		22 (13.8)	19 (11.9)	
Infiltrative	61 (24.0)	40 (15.7)		71 (27.6)	29 (11.3)		28 (17.5)	19 (11.9)	
Budding									
Low	79 (31.6)	106 (42.4)	0.021	76 (30.0)	107 (42.3)	<0.001	58 (37.2)	59 (37.8)	1.0
High	39 (15.6)	26 (10.4)		47 (18.6)	23 (9.1)		20 (12.8)	19 (12.2)	
Grade of differentiation									
Well (G1)	10 (3.7)	17 (6.3)	0.279	10 (3.7)	15 (5.5)	0.415	10 (6.0)	9 (5.4)	0.599
Moderate (G2)	97 (36.1)	95 (35.3)		95 (34.9)	91 (33.5)		54 (32.3)	55 (32.9)	
Poor (G3)	28 (10.4)	22 (8.2)		34 (12.5)	27 (9.9)		23 (13.8)	16 (9.6)	
Relapse									
No	148 (67.3)	45 (72.6)	0.536	93 (63.3)	98 (71.0)	0.169	37 (61.7)	85 (73.3)	0.124
Yes	72 (32.7)	17 (27.4)		54 (36.7)	40 (29.0)		23 (38.3)	31 (26.7)	

**VD**_**S**_ stroma-normalised vessel density (vessels per mm^2^), *p* p-value, *TS* thymidylate synthase. Pearson chi-square test was used for statistical analysis

^a^—to splenic flexure

### Identification of high stroma-normalised vessel density as a candidate treatment effect-predictive marker for 5-FU-based adjuvant therapy

To identify a VD-related marker associated with the benefit of adjuvant therapy, all six metrics were analysed with regard to the ability to define subgroups, showing distinct patterns of response to adjuvant treatment. All analyses were performed using unbiased median-based cut-offs to define ‘marker-high' and ‘marker-low' subgroups. Separate analyses were performed using TTR or OS as the endpoint.

**Fig. 2:**
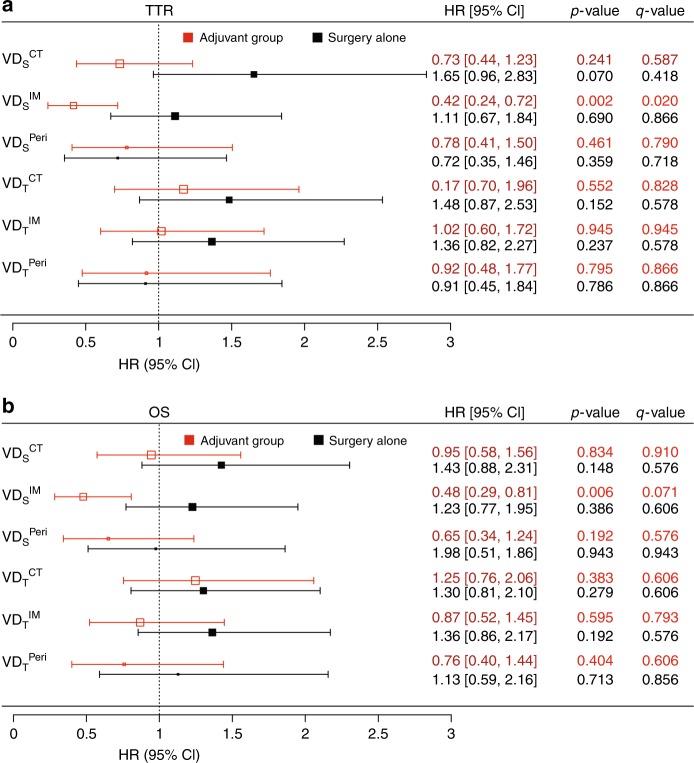
Associations between VD and survival rates in stage II/III colon cancer in the discovery cohort. Plot illustrating the hazard ratios (squares on the graph and values on the right) and 95% CI (whiskers and values in the square parentheses), derived from Cox-regression analyses, for TTR (**a**) and OS (**b**) in the adjuvant chemotherapy group (red) and surgery-alone group (black). *P*-values, adjusted for multiple testing, are shown as q-values

As shown in Fig. 2a, (red colour code), a clearly significant benefit of adjuvant therapy was observed in the (**VD**_**S**_^**IM**^)-high subgroup, using TTR as the endpoint. A similar pattern was observed when using OS as the endpoint (Fig. 2b, red colour code); however, the effect was less prominent, and the statistical significance lost, when correction for multiple testing was applied (Fig. 2, q-values). Nevertheless, the formal interaction test confirmed statistically significant interaction between treatment and the **VD**_**S**_^**IM**^ (p = 0.009) in the OS-based analyses. Significant benefit of adjuvant treatment was not observed in any of the other subgroups, neither in the TTR- nor OS-based analyses.

To analyse, if the associations of **VD**_**S**_^**IM**^ with TTR and OS in the adjuvant-treated group represent only effects of the markers on response to treatment, or also include associations between the marker and intrinsic aggressiveness of the disease, all **VD** metrics were analysed with regard to associations with outcomes in patients not receiving adjuvant therapy. No significant associations between the marker and TTR or OS were observed in these analyses (Fig. 2, black colour code).

MMR status has been reported to determine response to adjuvant therapy.^16^ Also in previous studies using material from the Nordic randomised clinical trial, MMR status and TS expression were linked to response to adjuvant therapy.^40,41^ Thus, to investigate the independence of the **VD**_**S**_^**IM**^ metric, the multivariable analyses, including MMR status and TS expression together with age, gender and clinical stage, were performed separately in the surgery-alone and adjuvant-treated groups. As shown in Table 2, tumour stage was the only factor associated with TTR in the surgery-alone group. However, in adjuvant-treated patients, only **VD**_**S**_^**IM**^ was independently associated with TTR (p = 0.017). Because **VD**_**S**_^**IM**^ was strongly correlated with the shape of the tumour-invasive border and budding (Table 1), multivariable analyses were also performed with these three metrics. As shown in Supplementary Table 3, configuration of the tumour border was associated with TTR in the surgery-alone group, while in the adjuvant-treated group, **VD**_**S**_^**IM**^ remained independently associated with TTR (*p* = 0.049).

**Table 2:** Multivariable analyses including VD_S_^IM^, MMR, TS, gender, age and stage for TTR in colon cancer patients treated with surgery alone (A) or with adjuvant chemotherapy (B)

Covariates	HR	95.0% CI for HR		*p*-value
		Lower	Upper	
A) Surgery-alone group				
VD_S_^IM^ (high vs low)	0.968	0.567	1.655	0.906
MMR status (proficient vs deficient)	1.762	0.747	4.157	0.196
TS expression (high vs low)	1.881	0.965	3.666	0.064
Gender (women vs men)	1.137	0.657	1.968	0.647
Age (66 years or older)	1.345	0.777	2.329	0.289
Stage (III vs II)	3.500	1.830	6.692	<0.001
B) Adjuvant chemotherapy group				
VD_S_^IM^ (high vs low)	0.473	0.256	0.873	0.017
MMR status (proficient vs deficient)	1.307	0.579	2.949	0.520
TS expression (high vs low)	1.279	0.635	2.578	0.491
Gender (women vs men)	0.785	0.430	1.434	0.430
Age (66 years or older)	0.750	0.427	1.319	0.318
Stage (III vs II)	1.583	0.845	2.965	0.152

*MMR* DNA mismatch-repair status, *TS* thymidylate synthase, *HR* hazard ratio, *CI* confidence interval. Cox-regression model was used for statistical analysis

These analyses thus identified high **VD**_**S**_ as a candidate biomarker for a stage II/III colon cancer subset benefiting from 5-FU-based adjuvant chemotherapy, with strongest data for **VD**_**S**_^**IM**^.

### Preliminary validation of biomarker potential of VD_S_^IM^

To preliminary validate these findings, similar analyses were performed on an independent population-based CRC collection of stage II/III colon cancers, which included cases that had received adjuvant 5-FU alone or with oxaliplatin as part of risk-stratified routine clinical care.

The design of the tissue microarray of this CRC collection (see Methods for details) allowed collection of **VD**_**S**_^**CT**^**, VD**_**S**_^**IM**^, **VD**_**T**_^**CT**^ and **VD**_**T**_^**IM**^ data (82, 85, 82 and 85 cases, respectively). Following staining and metric collection, the dichotomisations were performed using median-based cut-off. Clinico-pathological characteristics of the validation cohort and the **VD**_**S**_ metrics-low and -high groups are shown in Table 3, with no statistically significant difference observed, except a higher fraction of patients with age ≥ 66 years in **the VD**_**S**_^**IM**^ group. We also compared the validation cohort and the respective group of the discovery cohort (adjuvant-treated, stage II/III, having material from the invasive margin). As expected, because of the population-based nature of the validation cohort, it has a higher fraction of stage III tumours. Also, a higher fraction of patients with distal tumour location was observed (Supplementary Table 4).

**Table 3:** Associations between VD_S_ and clinico-pathological characteristics in the validation cohort

	Tumour centre			Invasive margin		
	VD_S_^CT^ number (percent)			VD_S_^IM^ number (percent)		
	Low	High	*p*	Low	High	*p*
Age (years)						
<66	21 (52.5)	19 (47.5)	0.659	25 (61.0)	16 (39.0)	0.040
≥66	20 (47.6)	22 (52.4)		17 (38.6)	27 (61.4)	
Sex						
Male	22 (51.2)	21 (48.8)	0.825	18 (40.9)	26 (59.1)	0.104
Female	19 (48.7)	20 (51.3)		24 (58.5)	17 (41.5)	
Tumour site						
Proximal^a^	17 (51.5)	16 (48.5)	0.822	17 (48.6)	18 (51.4)	0.897
Distal	24 (49.0)	25 (51.0)		25 (50.0)	25 (50.0)	
Stage						
II	6 (60.0)	4 (40.0)	0.500	6 (60.0)	4 (40.0)	0.476
III	35 (48.6)	37 (51.4)		36 (48.0)	39 (52.0)	
Relapse						
Yes	13 (59.1)	9 (40.9)	0.205	13 (56.5)	10 (43.5)	0.286
No	21 (42.9)	28 (57.1)		22 (43.1)	29 (56.9)	

**VD**_**S**_ stroma-normalised vessel density (vessels per mm^2^), *p* *p*-value, Pearson chi-square test used for statistical analysis

^a^—to splenic flexure

**Fig. 3:**
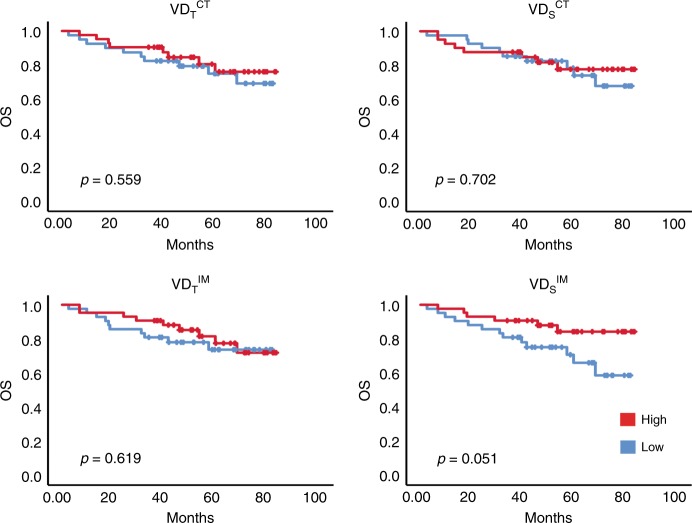
Associations between VD and survival rates in stage II/III colon cancer with adjuvant treatment in the validation cohort. Kaplan–Meier plots for **VD**_**S**_ -high (red) and -low (black) subgroups of adjuvant-treated patients in the validation cohort with OS as endpoints. Log-rank analyses were used for determination of *p*-values

The **VD** metrics were analysed for association with OS. Similar to the discovery cohort, no survival associations were detected for the **VD**_**T**_^**CT**^**, VD**_**T**_^**IM**^ and **VD**_**S**_^**CT**^. Instead, the **VD**_**S**_^**IM**^ separated patients into groups with different survival rates, similar to the observation in the discovery cohort, although the statistical significance of this observation was marginal (*p* = 0.051, log-rank test) (Fig. 3).

Together, these analyses, although not yielding statistically significant results, support the finding from the analyses of the randomised-trial-derived discovery cohort, including indications that the stroma-normalised vessel-density metric performs better as a biomarker than the total vessel-density score.

## Discussion

This study identifies high stroma-normalised VD in the invasive margin as a candidate marker for identification of patients benefiting of adjuvant chemotherapy in stage II–III colon cancer.

In spite of a history of almost 30 years, the studies on vessel density as biomarkers have not been translated to any marker of clinical utility.^42^ It can be noted that most of these studies have focused on prognostic relevance. Already more than 10 years ago, meta-analyses of breast and colorectal cancer studies identified vessel density as a prognostic factor.^43,44^ However, many issues, including the remaining inconsistencies in findings and lack of assay standardisation have hampered the field. Recent studies have introduced other vessel-related metrics than vessel density to explore associations with benefit of chemotherapy. This includes e.g. studies linking pericyte status to response to chemotherapy (reviewed in refs. ^45–47^). In this context, this study has some special features; the study is emphasising a candidate marker of predictive, not prognostic relevance, the discovery cohort is composed of a population derived from a randomised controlled trial and the vessel metric is integrating normalisation to stroma and a spatial restriction to the invasive margin. Obviously, independent validation of the key finding in tumour collections from other randomised trials of adjuvant therapy in colorectal trials is a key topic for future studies. Future studies should also explore the relevance of the novel metric in other tumour types, including breast cancer, where earlier studies with other vessel-density metrics have failed to detect associations between vessel density and benefit of chemotherapy.^48^

Identification of robust and strong markers predicting benefit of adjuvant chemotherapy is recognised as a key challenge in translational colorectal cancer research.^11,49,50^ The most mature marker is deficiency in a mismatch repair, which is associated with lack of benefit in high-risk stage II CRC.^16^ A mesenchymal gene expression profile (CMS4) has also been linked to reduced benefit.^51^ Candidate markers associated with benefit of adjuvant therapy include thymidylate synthase (TS) and high Oncotype-DX risk score.^41,52^ As shown in Table 2, **VD**_**S**_^**IM**^ remained significantly associated with TTR in multivariable analyses, also including TS and mismatch-repair status. This suggests that the predictive capacity of **VD**_**S**_^**IM**^ is related to other biological mechanisms than those underlying the potential response-predictive capacity of TS and mismatch-repair status. This should be further analysed in analyses of other well-annotated cohorts.

The tumour collection of the discovery cohort is derived from a randomised trial, which makes it suitable for the identification of treatment-predictive biomarkers. For the validation cohort, a recent (2010–2014) population-based tumour collection in which patients were treated according to current ESMO guidelines was selected. Some limitations of the study are recognised. Concerning treatment regimens, it is noted, firstly, that the treatments used in the discovery cohort differ from present standards, and secondly, that the treatments used in the validation and discovery cohorts differ. Furthermore, limitations regarding the procedures for lymph-node dissection of the discovery cohort prevented stringent stage-specific analyses. Additional studies in other well-defined cohorts are therefore highly motivated.

Concerning analytical procedures, the study relies on digital scoring which should increase stringency and possibly also allow more quantitative scoring. Notably, continued development of the candidate biomarker towards clinical utility will require extensive standardisation efforts and additional work on selection of optimal cut-offs. From an analytical perspective, it should be noted that the preliminary data from the validation cohort were derived from the analyses of two representative TMA cores, each 1 mm in diameter, from the tumour centre and invasive margin, whereas the discovery cohort used large blocks for analyses. Future optimisation studies should address the issue of ‘inside-case' heterogeneity and how this can best be addressed.

The analyses of the surgery-alone group showed that in this population, **VD**_**S**_^**IM**^ was not significantly associated with TTR or OS (Fig. 2). This suggests that **VD**_**S**_^**IM**^ is related to the benefit of adjuvant therapy, without being coupled to the natural course or intrinsic aggressiveness of the disease. This notion is also supported by the fact that **VD**_**S**_^**IM**^ is not significantly associated with stage in the discovery cohort (Table 1), or in the validation cohort (Table 3). Notably, **VD**_**S**_^**IM**^ shows in the discovery cohort an inverse association with poor prognostic factors, such as budding and infiltrative growth (Table 2).

The underlying biology should be explored in future mechanistic studies. One possibility to consider in such efforts is that the vascular phenotype of the primary tumour is a proxy for a chemo-resistant population, in agreement with earlier experimental studies which have identified perivascular niches that host and support special cancer cell populations.^21,23,24^ Future studies should also investigate if the high stroma-normalised VD in the invasive margin is associated with certain driver mutations, or gene expression profiles, linked to the recently defined molecular subtypes of colon cancer.

From a general vascular biomarker perspective, the study confirms that increasing biological content in VD measurements, as done here by considering different compartments and normalisation to stroma abundance, can increase performance of VD-related markers. This finding should inspire to continued efforts, in many tumour types, of in-depth vascular profiling towards the ultimate goal of developing a predictive vascular biomarker of clinical utility.

## Supplementary information

Supplementary Figures and Tables

## Data Availability

The data sets used and analysed during this study are available from the corresponding author.
