# Supplementary material for: Stroma-normalised vessel density predicts benefit from adjuvant fluorouracil-based chemotherapy in patients with stage II/III colon cancer
Source: Br J Cancer. 2019 Jul 10;121(4):303–11. doi: 10.1038/s41416-019-0519-1 (PMC6738077; doi:10.1038/s41416-019-0519-1)
Supplement: Supplementary file 1 — Supplementary Figures and Tables [file 41416_2019_519_MOESM1_ESM.docx]

**Supplementary Figures.**

**Supplementary Figure 1.**

**A. Schematic illustration of the region/compartment selection on the section of the tumor sample (discovery cohort).**

**B. Venn diagram representing the relationship between cases available from the discovery cohort for the analysis of the three different compartments: tumor center, invasive margin and peritumoral stroma.**

**
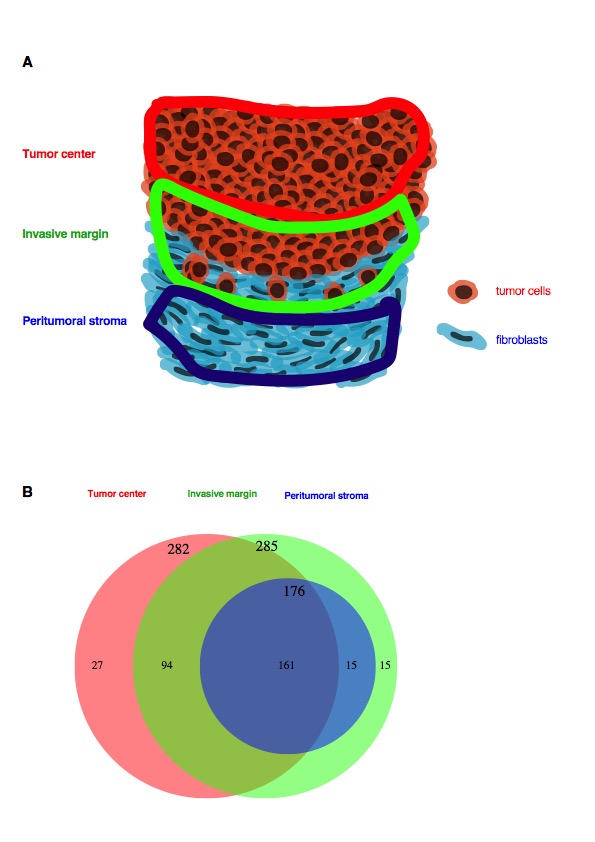
**

**Supplementary Figure 2. Associations between adjuvant chemotherapy and survival rates in the discovery cohort**. Kaplan-Meier plots for TTR (left) and OS (right) in the surgery alone (black) and adjuvant chemotherapy (red) patients. Log-rank test was used for statistical analysis.

**Supplementary Figure 3.** **Comparison between VD_T_ and VD_S_ in the different tumor regions in the discovery cohort.** Box plots illustrate median levels (horizontal line), 1^st^ and 3^rd^ quartiles (box) and 1,5 IQR-whiskers of the **VD_T_ *and* VD_S_** in three tumor regions: tumor center (red), invasive margin (green) and peritumoral stroma (blue). Mann-Whitney U test was used to evaluate differences between groups.

**Supplementary Figure 4.** **Correlations between VD_T_ and VD_S_ in the different tumor compartments in the discovery cohort*.*** Spearman rank correlation test used and coefficient r is shown.

******

**SUPPLEMENTARY. TABLES.**

**Supplementary Table 1. Clinico-pathological characteristics of the three populations, used for the determination of vessel density in tumor center, invasive margin and peritumoral stroma. Discovery cohort.**

|  | **Tumor center** | **Invasive margin** | **Peritumoral stroma** | **p-value** |
| --- | --- | --- | --- | --- |
| **Age (Years)** |  |  |  |  |
| < 66 | 122  (43.3) | 130  (45.6) | 83  (47.2) | 0.699 |
| ≥ 66 | 160  (56.7) | 155  (54.4) | 93  (52.8) |  |
| **Sex** |  |  |  |  |
| Male | 147  (52.1) | 143  (50.2) | 87  (49.4) | 0.829 |
| Female | 135  (47.9) | 142  (49.8) | 89  (50.6) |  |
| **Tumor Site** |  |  |  |  |
| Proximal^a^ | 149  (52.8) | 163  (57.2) | 97  (55.1) | 0.581 |
| Distal | 133  (47.2) | 122  (42.8) | 79  (44.9) |  |
| **Mismatch repair status** |  |  |  |  |
| MMR proficient | 226  (85.9) | 216  (81.2) | 127  (78.4) | 0.116 |
| MMR deficient | 37  (14.1) | 50  (18.8) | 35  (21.6) |  |
| **TS expression** |  |  |  |  |
| High | 213  (75.5) | 217  (76.1) | 137  (77.8) | 0.849 |
| Low | 69  (24.5) | 68  (23.9) | 39  (22.2) |  |
| **Stage** |  |  |  |  |
| II | 117  (41.5) | 117  (41.1) | 73  (41.5) | 0.993 |
| III | 165  (58.5) | 168  (58.9) | 103  (58.5) |  |
| **Invasive border configuration** |  |  |  |  |
| Pushing | 95  (37.4) | 99  (38.5) | 72  (45.0) | 0.257 |
| Intermediate | 58  (22.8) | 58  (22.6) | 41  (25.6) |  |
| Infiltrative | 101  (39.8) | 100  (38.9) | 47  (29.4) |  |
| **Budding** |  |  |  |  |
| Low | 185  (74.0) | 183  (72.3) | 117  (75.0) | 0.824 |
| High | 65  (26.0) | 70  (27.7) | 39  (25.0) |  |
| **Grade of differentiation** |  |  |  |  |
| Not evaluated | 13  (4.6) | 13  (4.6) | 9  (5.1) | 0.861 |
| Well (G1) | 27  (9.6) | 25  (8.8) | 19  (10.8) |  |
| Moderate (G2) | 192  (68.1) | 186  (65.3) | 109  (61.9) |  |
| Poor (G3) | 50  (17.7) | 61  (21.4) | 39  (22.2) |  |

Abbreviations: p, p-value; TS, thymidylate synthase. Pearson Chi-Square test used for statistical analysis.

^a^ -to splenic flexure.

**Supplementary Table 2. Clinico-pathological characteristics of the three populations, sub-divided by treatment, used for the determination of vessel density in tumor center, invasive margin and peritumoral stroma**

|  | **Tumor center**  **n=282** | | | **Invasive margin**  **n=285** | | | **Peritumoral stroma**  **n=176** | | |
| --- | --- | --- | --- | --- | --- | --- | --- | --- | --- |
|  | **No-adj** | **Adj** | p | **No-adj** | **Adj** | p | **No-adj** | **Adj** | p |
| **Age (Years)** |  |  |  |  |  |  |  |  |  |
| < 66 | 59 (41.3) | 63  (45.3) | 0.491 | 65 (43.3) | 62  (48.1) | 0.415 | 43 (47.3) | 40  (47.1) | 0.979 |
| ≥ 66 | 84  (58.7) | 76  (54.7) |  | 85  (56.7) | 70  (51.9) |  | 48  (52.7) | 45  (52.9) |  |
| **Sex** |  |  |  |  |  |  |  |  |  |
| Male | 73  (51.0) | 74  (53.2) | 0.713 | 75 (50.0) | 67  (49.6) | 0.950 | 44 (48.4) | 43  (50.6) | 0.767 |
| Female | 70  (49.0) | 65  (46.8) |  | 75 (50.0) | 68  (50.4) |  | 47  (51.6) | 42  (49.4) |  |
| **Tumor Site** |  |  |  |  |  |  |  |  |  |
| Proximal^a^ | 81  (56.5) | 68  (48.9) | 0.194 | 90 (60.0) | 73  (54.1) | 0.313 | 54 (59.3) | 43  (50.6) | 0.243 |
| Distal | 62  (43.4) | 71  (51.1) |  | 60  (40.0) | 62  (45.9) |  | 37  (40.7) | 42  (49.4) |  |
| **Mismatch repair status** |  |  |  |  |  |  |  |  |  |
| MMR proficient | 114  (86.4) | 112  (85.5) | 0.840 | 115 (82.7) | 101  (79.5) | 0.504 | 66 (80.5) | 61  (76.3) | 0.512 |
| MMR deficient | 18  (13.6) | 19  (14.5) |  | 24  (17.3) | 26  (20.5) |  | 16  (19.5) | 19  (23.8) |  |
| **TS expression** |  |  |  |  |  |  |  |  |  |
| High | 105  (73.4) | 108  (77.7) | 0.404 | 112 (74.4) | 105  (77.8) | 0.538 | 71 (78.0) | 66  (77.6) | 0.952 |
| Low | 38  (26.6) | 31  (22.3) |  | 38  (25.3) | 30  (22.2) |  | 20  (22.0) | 19  (22.4) |  |
| **Stage** |  |  |  |  |  |  |  |  |  |
| II | 64  (44.8) | 53  (38.1) | 0.259 | 64 (42.7) | 53  (39.3) | 0.559 | 43 (47.3) | 30  (35.3) | 0.108 |
| III | 79  (55.2) | 86  (61.9) |  | 86  (57.3) | 82  (60.7) |  | 48  (52.7) | 55  (64.7) |  |
| **Invasive border configuration** |  |  |  |  |  |  |  |  |  |
| Pushing | 57  (40.4) | 47  (34.6) | 0.592 | 59 (40.4) | 48  (36.6) | 0.698 | 44  (50.0) | 34  (41.0) | 0.211 |
| Intermediate | 31  (22.0) | 34  (25.0) |  | 30  (20.5) | 32  (24.4) |  | 23  (26.1) | 19  (22.9) |  |
| Infiltrative | 53  (37.6) | 55  (40.4) |  | 57  (39.0) | 51  (38.9) |  | 21  (23.9) | 30  (36.1) |  |
| **Budding** |  |  |  |  |  |  |  |  |  |
| Low | 106  (76.3) | 96  (72.7) | 0.505 | 103 (71.5) | 94  (74.0) | 0.646 | 65  (75.6) | 61  (76.3) | 0.920 |
| High | 33  (23.7) | 36  (27.3) |  | 41  (28.5) | 33  (26.0) |  | 21  (24.4) | 19  (23.8) |  |
| **Grade of differentiation** |  |  |  |  |  |  |  |  |  |
| Well (G1) | 13  (9.6) | 14  (10.5) | 0.851 | 12 (8.4) | 13  (10.1) | 0.791 | 10  (11.5) | 9  (11.3) | 0.964 |
| Moderate (G2) | 96  (70.6) | 96  (72.2) |  | 97  (67.8) | 89  (69.0) |  | 56  (64.4) | 53  (66.3) |  |
| Poor (G3) | 27  (19.9) | 23  (17.3) |  | 34  (23.8) | 27  (20.9) |  | 21  (24.1) | 18  (22.5) |  |
| **Relapse** |  |  |  |  |  |  |  |  |  |
| No | 99  (69.2) | 94  (67.6) | 0.799 | 100  (66.7) | 91  (67.4) | 0.900 | 64  (70.3) | 58  (68.2) | 0.870 |
| Yes | 44  (30.8) | 45  (32.4) |  | 50  (33.3) | 44  (32.6) |  | 27  (29.7) | 27  (31.8) |  |

Abbreviations: p, p-value; TS, thymidylate synthase. Pearson Chi-Square test used for statistical analysis.

^a^ -to splenic flexure.

**Supplementary Table 3. Multivariable analyses including VD_S_^IM^, Invasive border configuration (Pushing vs Intermediate vs Infiltrative) and budding (High vs Low) for TTR in colon cancer patients treated with surgery alone (A) or with 5-FU-based chemotherapy (B).**

| 1. ***Surgery alone group*** | | | | |
| --- | --- | --- | --- | --- |
| Covariates | HR | 95.0% CI for HR | | p-value |
|  |  | Lower | Upper |  |
| **VD_S_^IM^ (high vs low)** | 1.447 | 0.816 | 2.565 | 0.206 |
| Invasive border configuration | 1.426 | 1.002 | 2.030 | 0.048 |
| Budding | 0.829 | 0.430 | 1.601 | 0.577 |
| 1. ***Adjuvant chemotherapy group*** | | | | |
| Covariates | HR | 95.0% CI for HR | | p-value |
|  |  | Lower | Upper |  |
| **VD_S_^IM^ (high vs low)** | 0.515 | 0.266 | 0.997 | 0.049 |
| Invasive border configuration | 1.227 | 0.804 | 1.873 | 0.343 |
| Budding | 1.375 | 0.705 | 2.681 | 0.350 |

Abbreviations: HR, hazard ratio; CI, confidence interval. Cox-regression model used for statistical analysis.

**Supplementary Table 4. Comparison of the clinico-pathological characteristics in the discovery and the validation cohort, among cases, used to generate stroma-normalized vessel density score at the invasive margin (VD_S_^IM^).**

|  | **Discovery cohort**  **N=135** | **Validation cohort**  **N=85** | **p-value** |
| --- | --- | --- | --- |
| **Age (Years)** |  |  |  |
| < 66 | 65  (48.1) | 41  (48.2) | 0.990 |
| ≥ 66 | 70  (51.9) | 44  (51.8) |  |
| **Sex** |  |  |  |
| Male | 68  (50.4) | 44  (51.8) | 0.950 |
| Female | 67  (49.6) | 41  (48.2) |  |
| **Tumor Site** |  |  |  |
| Proximal^a^ | 73  (54.1) | 35  (41.2) | 0.085 |
| Distal | 62  (45.9) | 50  (58.8) |  |
| **Stage** |  |  |  |
| II | 53  (39.3) | 10  (11.8) | <0.001 |
| III | 82  (60.7) | 75  (88.2) |  |
| **Relapse** |  |  |  |
| Yes | 44  (32.6) | 23  (27.1) | 0.945 |
| No | 91  (67.4) | 51  (60.0) |  |
| Missing data |  | 11  (12.9) |  |

Abbreviations: p-value; Pearson Chi-Square test used for statistical analysis.

^a^ -to splenic flexure.
